# Selective Valve Removal for Melody Valve Endocarditis: Practice Variations in a Multicenter Experience

**DOI:** 10.1007/s00246-021-02801-z

**Published:** 2021-12-11

**Authors:** Arpine Davtyan, Peter W. Guyon, Hannah R. El-Sabrout, Reid Ponder, Nanda Ramchandar, Rachel Weber, Wagih Zayed, Kanishka Ratnayaka, John J. Nigro, John W. Moore, Holly Bauser-Heaton, Laith Alshawabkeh, Ryan R. Reeves, Daniel Levi, Jamil Aboulhosn, Henri Justino, John Bradley, Howaida G. El-Said

**Affiliations:** 1 Cardiology, Rady Children’s Hospital, University of California San Diego, 3020 Children’s Way, MC 5004, San Diego, CA 92123 USA; 2 Mattel Children’s Hospital, University of California Los Angeles, Los Angeles, CA USA; 3 Children’s Healthcare of Atlanta, Emory University, Atlanta, GA USA; 4 Texas Children’s Hospital, Baylor University, Houston, TX USA; 5 Infectious Diseases, Rady Children’s Hospital, University of California San Diego, San Diego, CA USA; 6 Cardiothoracic Surgery, Rady Children’s Hospital, University of California, San Diego, CA USA; 7 Division of Cardiovascular Medicine, University of California, San Diego, USA

**Keywords:** Endocarditis, Congenital heart disease, Transcatheter pulmonary valve, Melody valve

## Abstract

Guidelines for management of *Melody* transcatheter pulmonary valve (TPV) infective endocarditis (IE) are lacking. We aimed to identify factors associated with surgical valve removal versus antimicrobial therapy in *Melody* TPV IE. Multicenter retrospective analysis of all patients receiving *Melody* TPV from 10/2010 to 3/2019 was performed to identify cases of IE. Surgical explants versus non-surgical cases were compared. Of the 663 *Melody* TPV implants, there were 66 cases of IE in 59 patients (59/663, 8.8%). 39/66 (59%) were treated with IV antimicrobials and 27/66(41%) underwent valve explantation. 26/59 patients (44%) were treated medically without explantation or recurrence with average follow-up time of 3.5 years (range:1–9). 32% of Streptococcus cases, 53% of MSSA, and all MRSA cases were explanted. 2 of the 4 deaths had MSSA. CART analysis demonstrated two important parameters associated with explantation: a peak echo gradient ≥ 47 mmHg at IE diagnosis(OR 10.6, *p* < 0.001) and a peak echo gradient increase of > 24 mmHg compared to baseline (OR 6.7, *p* = 0.01). Rates of explantation varied by institution (27 to 64%). In our multicenter experience, 44% of patients with *Melody* IE were successfully medically treated without valve explantation or recurrence. The degree of valve stenosis at time of IE diagnosis was strongly associated with explantation. Rates of explantation varied significantly among the institutions.

**Supplementary Information:**

The online version contains supplementary material available at 10.1007/s00246-021-02801-z.

## Introduction

Early reports of transcatheter pulmonary valve (TPV) implantation using the *Melody TPV* (Medtronic, Minneapolis, MN) have demonstrated good hemodynamic and clinical outcomes [1]. Infective endocarditis (IE) of the valve has been identified as a potential adverse event [2–4]. Additional studies have confirmed the incidence of IE in *Melody* TPV is significant and is a potential threat to valve function and long-term outcomes [5–7].

The consequences of IE after *Melody* TPV can be serious and are the subject of active research. A recent systematic review reported mortality from *Melody* TPV endocarditis might be as high as 8.7% [7]. Up to 45% of patients with *Melody* TPV endocarditis undergo valve explantation and up to 12% undergo transcatheter intervention [7]. There are patients who do not require surgery or catheter-based re-intervention and are successfully treated for IE medically [4, 6, 7].

Most studies examining IE after *Melody* TPV implantation have focused on identifying risk factors associated with developing endocarditis [8–10]. This retrospective study aims to identify the factors associated with the need for surgical explantation as opposed to those associated with successful antimicrobial therapy alone. Identifying such factors will help determine which patients require surgical removal and which may benefit solely from antimicrobial therapy.

## Methods

This is a retrospective chart review of all patients who received transcatheter *Melody* valves from October 2010 to March 2019 at four congenital heart centers (Rady Children’s Hospital/University of California San Diego [UCSD], Mattel Children’s Hospital/University of California Los Angeles [UCLA], Children’s Healthcare of Atlanta/Emory University, and Texas Children’s Hospital/Baylor University). This multicenter retrospective study was approved by all four respective Institutional Review Boards (IRB). A waiver of informed consent was granted by the respective IRBs. The study period initiation (October 2010) coincides when the four centers began performing *Melody* TPV implantation. March 2019 was chosen as the final date of *Melody* TPV implantation to allow for all patients to have at least one year of post-procedure follow-up. We included any patient (all ages) who received *Melody* TPV during the study period. We excluded patients who underwent *Melody* TPV placement in locations other than the pulmonary valve position.

The electronic medical record [progress notes, echocardiograms, and microbiology/laboratory results (after *Melody* TPV placement)] of each patient was reviewed to identify patients who developed IE. The diagnosis of IE was defined as any bloodstream infection treated with intravenous (IV) antimicrobials for longer than 4 weeks after *Melody* TPV insertion and presumed to be related to the *Melody* TPV. In most cases this included either a clinical presentation of infection (e.g., fever, elevated inflammatory markers) or positive blood culture with or without *Melody* TPV dysfunction/vegetation. Culture negative endocarditis was diagnosed when there was a clinical presentation of infection (e.g., fever, elevated inflammatory markers) with negative blood cultures but with new Melody TPV dysfunction (either stenosis or regurgitation) and/or a vegetation. Presence of a vegetation on the valve was not required for diagnosis as vegetations can be difficult to visualize by echocardiogram, particularly if small and in patients with poor echocardiographic windows.

Baseline patient characteristics including sex, weight, congenital heart disease, and surgical history were obtained from review of the electronic medical record. *Melody* TPV implantation details were obtained and included age and weight at implantation, valve size, right ventricular outflow tract (RVOT) landing zone (native, conduit, or bioprosthetic), pre-stent number, and hemodynamic data (e.g., baseline gradient and post-procedural gradient across the *Melody* TPV). Other data collected on all patients included time from TPV implantation to development of IE, the causative organism, surgical and medical treatment outcomes [successful treatment with IV antimicrobials without valve removal, surgical removal, transcatheter intervention, death], and duration of follow-up. Echocardiographic evaluations of the valve from implantation to latest follow-up were also obtained. All patients were followed until April 2021.

Cases of *Melody* TPV IE that were surgically explanted were retrospectively compared against those which were not explanted to identify factors associated with removal versus successful medical treatment. A univariate regression model was used to evaluate each potential factor’s effect on the outcome (*SPSS*, IBM Corporation, Armonk, NY). The small sample size precluded multivariate regression analysis. Descriptive data are presented as numbers with percentages or medians (Q1–Q3). Statistical significance was defined as *p* < 0.05. Classification and regression tree (CART) analysis was used to understand the effect of different variables on *Melody* TPV explantation using R (R Core Team [2013]. R: A language and environment for statistical computing. R Foundation for Statistical Computing, Vienna, Austria.)

## Results

### Baseline Characteristics and Outcomes

**Fig. 1:**
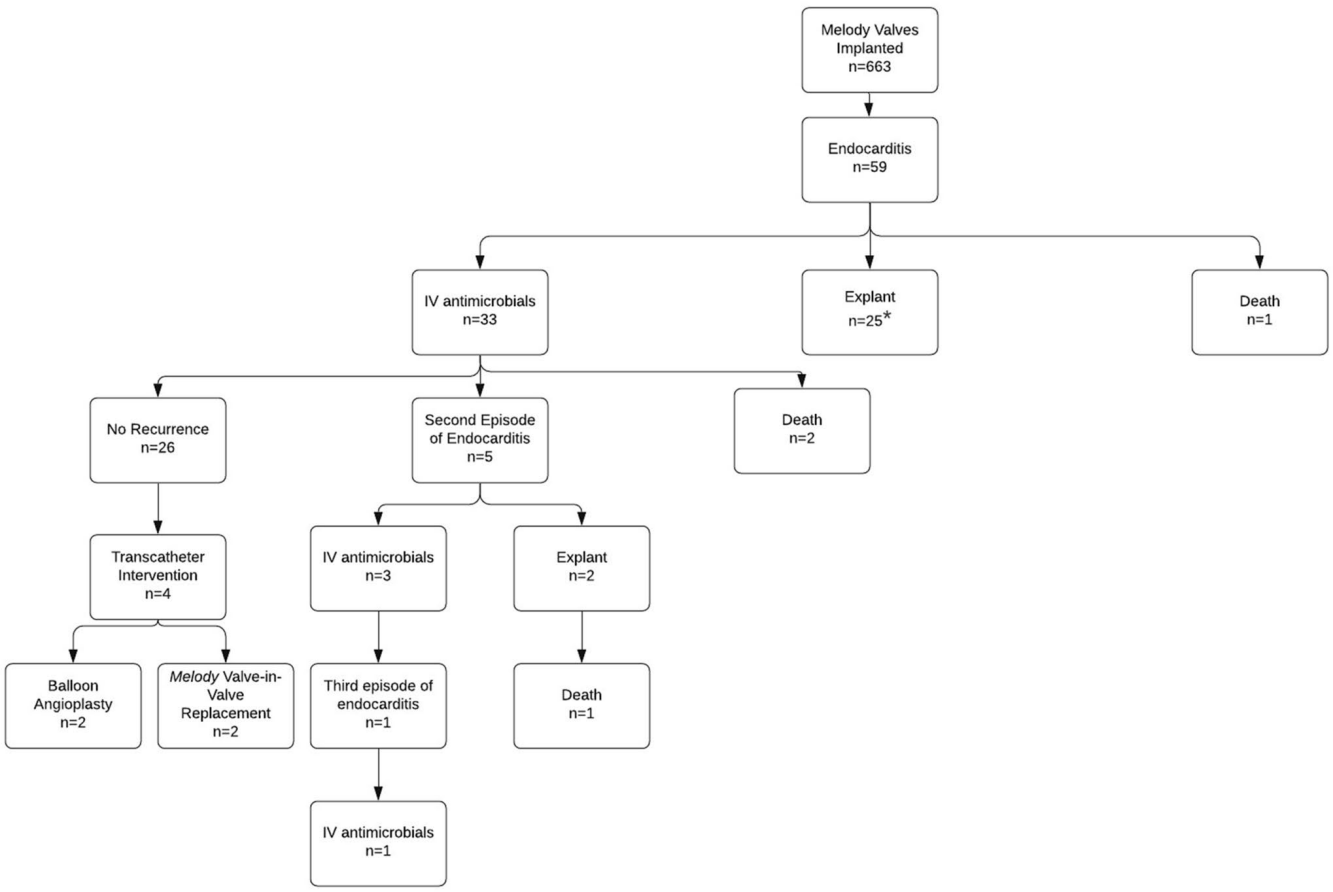
Outcomes after Melody Valve Infective Endocarditis. *One of these patients had MSSA endocarditis and removal of the *Melody* valve. Three years later, the patient had a second Melody valve implanted within his homograft. The patient developed MSSA endocarditis about two months after placement of the second valve. This was treated with IV antimicrobials. Three years later the patient had a third episode of MSSA endocarditis and the valve was explanted

A total of 663 *Melody* TPV were implanted in the four centers during the study period (October 2010 to March 2019). 59 patients (59/663, 8.8%) developed endocarditis. Some patients had recurrence and there were a total of 66 cases of endocarditis. (Fig. 1). Of the 66 cases of endocarditis, 39 (59%) were treated with IV antimicrobials without surgical explantation and 27 cases (41%) had the valve explanted with concurrent antimicrobial therapy. Two patients required transcatheter balloon valvuloplasty as part of their acute management. Two patients had transcatheter valve-in-valve replacements 9.2 and 19.5 months after completing treatment for IE without recurrence of IE.

Overall, the baseline characteristics were similar between the explanted and not explanted groups (Table 1). There was a statistically significant difference in age between the two groups. The explanted group had a median age of 17 years, while the non-explanted group had a median age of 22 years (*p* = 0.01). There was a higher proportion of adults (aged 21 or older) in the non-explanted group compared to the explanted group, (*p* = 0.03).

**Table 1:** Baseline characteristics and risk factors for melody valve explantation

	MV Not Explanted (*n* = 39)	MV Explanted (*n* = 27)	*P*-value
Age (y), median (Q1–Q3)	22 (17–33)	17 (14–22)	**0.01**
Adult (≥ 21y), *n* (%)	21 (54%)	7 (26%)	**0.03**
Male Sex, *n* (%)	26 (67%)	16 (59%)	0.54
Underlying cardiac diagnosis, *n* (%)			
TOF	17 (44%)	13 (48%)	0.45
Truncus arteriosus	5 (13%)	4 (15%)	0.95
DORV or TGA	7 (18%)	4 (15%)	0.70
Left heart disease s/p Ross	8 (20%)	3 (11%)	0.36
Pulmonary atresia/stenosis	2 (5%)	3 (11%)	0.26
RVOT Type, *n* (%)			
Native	1 (3%)	1 (4%)	0.82
Conduit	25 (64%)	18 (67%)	0.27
Bioprosthetic	13 (33%)	8 (29%)	0.78
Peak RVOT gradient (echo) at time of endocarditis diagnosis ≥ 47 mmHg, N (%)	7 (21.2%)	20 (74.1%)	** < 0.001**
Increase in RVOT gradient (echo) > 24 mmHg from baseline, N (%)	4 (12.1%)	13 (48.1%)	**0.01**
RVOT Gradient (echo) at Diagnosis, median(Q1–Q3)	29 mmHg (26–46)	64 mmHg (47–72)	**0.04**
Change in RVOT gradient (echo) from baseline, median(Q1–Q3)	8 mmHg (-4–13)	22 mmHg (2–40)	**0.04**
Vegetations (echo), N (%)	12 (31.6%)	12 (44.4%)	0.32
Vegetations (echo) and Staphylococcal species, N (%)	3 (7.7%)	5 (18.5%)	0.20
Staphylococcal species, N (%)	7 (17.9%)	10 (37%)	0.07
Streptococcal species, N (%)	17 (43.6%)	8 (29.6%)	0.26
Time to endocarditis, median(Q1–Q3) (range)	2.3 yr (1.2–4.6)	2.8 yr (1.7–5.1)	0.76
Residual RVOT Gradient (at time of TPV implant), median (Q1–Q3)	10 mmHg (8–14)	12 mmHg (8–16)	0.08
Number of pre-stents, median(Q1–Q3)	1.0 (0–1.5)	1.0 (0.5–2)	0.67

**Fig. 2:**
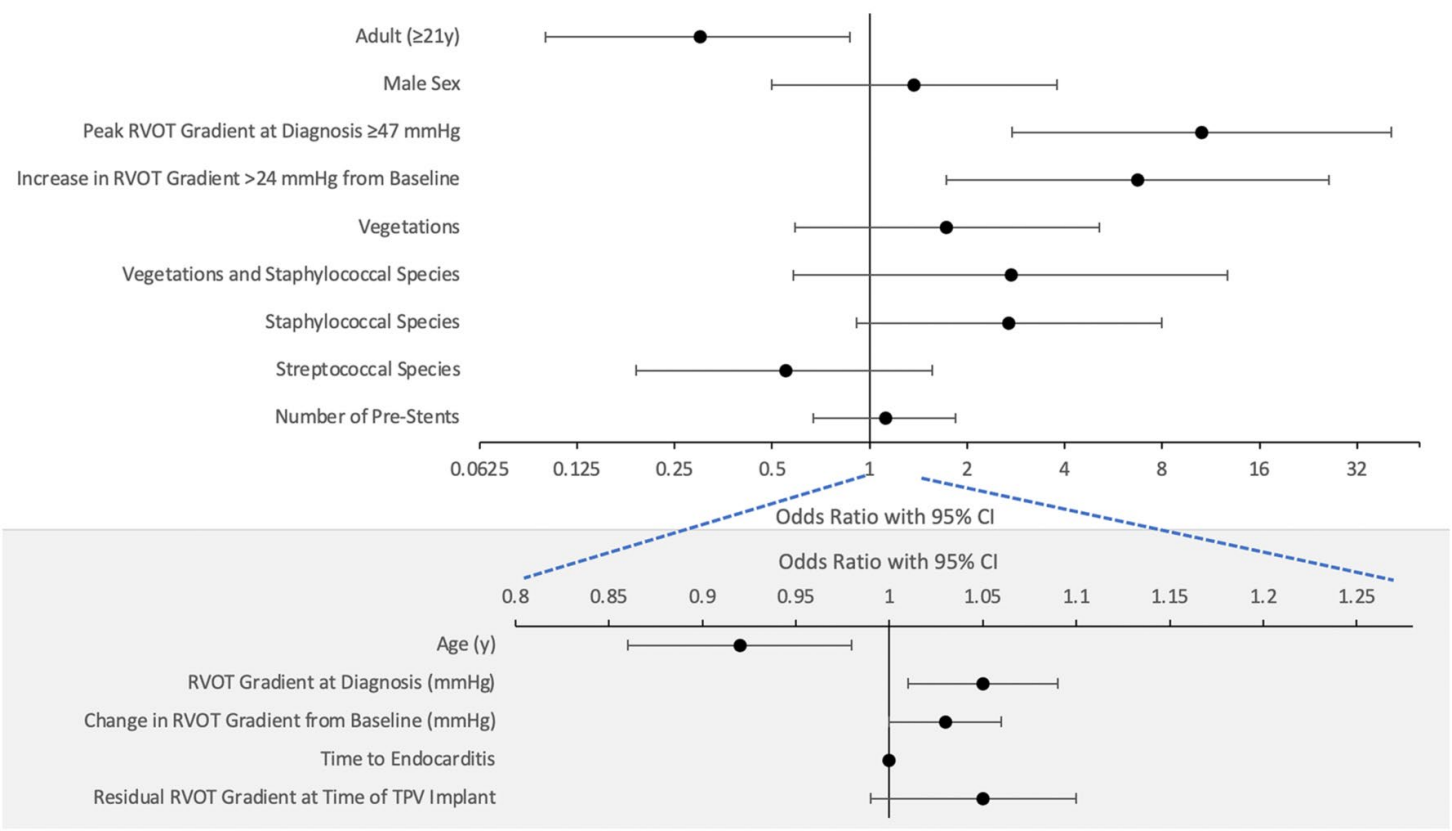
Forest plot of odds ratios and the 95% confidence interval for baseline characteristics and risk factors for *Melody* Valve explantation

Of the 59 patients initially diagnosed with IE, 26 (44%) were treated with IV antimicrobials without explantation or recurrence with an average follow-up duration of 3.5 years (range: 1 to 9 years). 33/59 patients were initially treated with IV antimicrobials alone. Six patients had recurrence. Four of the six patients had one additional episode of IE after the original diagnosis. In these four patients, the second episode of IE was caused by a different organism than the first episode. Two of the six patients had two additional episodes (Fig. 1). All endocarditis episodes in the first patient were caused by different species of streptococcus. In the second patient, all three episodes were caused by methicillin-sensitive *Staphylococcus aureus* (MSSA). Recurrent episodes were more than one year after initial IV antimicrobial treatment. None of the patients with multiple episodes of endocarditis had a known diagnosis of immunodeficiency. Further details regarding the patients with recurrent episodes of endocarditis are included with the supplemental materials (Supplemental Information: Infective Endocarditis Recurrences) (Fig. 2).

### Causes of Endocarditis and Mortalities

Streptococcus species were the most commonly identified organisms (38%); 32% of these cases resulted in valve explantation (Table 2). The next most common organism was *Staphylococcus aureus* in 26% [23% MSSA and 3% methicillin-resistant *Staphylococcus aureus* (MRSA)]. Fifty-three percent of the MSSA cases and all the MRSA cases resulted in explantation. Two of the four deaths in the cohort were attributed to MSSA endocarditis. The next most common category of endocarditis was “culture negative” endocarditis at 15% (40% of which were explanted). The third *Melody* TPV endocarditis-related death was in this group. The next most common cause of endocarditis was the HACEK organisms at 9% (50% explanted). The fourth mortality was in a patient with *Cardiobacterium hominis* endocarditis*,* although the cause of death was secondary to nosocomial fungemia more than 100 days after initial presentation. Four patients out of the total of 59 patients with IE (6.7%) died during treatment (Table 3). One additional case resulted in mortality more than 1.4 years after successful treatment of IE and was unrelated to the *Melody TPV* (end-stage renal disease).

**Table 2:** Causative organisms

Organism	Number of Cases, *N* (% of total)	Explanted, *N* (%)	Death, *N* (%)
Total	66	27/66 (41%)	4/66 (6%)
Streptococcal species	25 (38%)	8/25 (32%)	–
Staphylococcal species			
MSSA	15 (23%)	8/15 (53%)	2/15 (13%)
MRSA	2 (3%)	2/2 (100%)	
Culture negative/ unknown	10 (15%)	4/10 (40%)	1/10 (10%)
HACEK organisms	6 (9%)	3/6 (50%)	1/6 (16%)
Enterococcus species	3 (5%)	0/3 (0%)	–
Bartonella henselae	2 (3%)	1/2 (50%)	–
Other bacteria	2 (3%)	0/2 (0%)	–
Fungus	1 (1%)	1/1 (100%)	–

**Table 3:** Mortalities

Patient	Time from *Melody* implantation to endocarditis (years)	Organism	Time from diagnosis of IE to death (days)	Cause of mortality and other details
1	2.1	Culture negative	1	Erroneously diagnosed with pelvic inflammatory disease at an outside hospital and later presented in shock with severe pulmonary stenosis and severe right ventricular (RV) dysfunction Died secondary to RV and subsequent left ventricular failure
2	2.7	MSSA	4	Died secondary to septic shock with multiorgan failure Surgical risk of immediate valve explantation deemed too high due to tenuous hemodynamic status
3	5	MSSA	4	Remote prior history of mitral valve endocarditis (organism unknown) before *Melody TPV* implantation Died secondary to septic shock and multiorgan failure Surgical risk of immediate valve explantation deemed too high due to tenuous hemodynamic status
4	7.5	*Cardiobacterium hominis*	101	Treated for *Haemophilus influenzae* endocarditis 5 years prior Valve removal 7 days after presentation due to persistent stenosis and pancytopenia During the surgery for *Melody* removal also underwent successful replacement of his ventricular septal defect patch and the entire aortic root which were also infected Died secondary to fungemia, osteomyelitis, and multiple thrombi

### Valve Explantation

**Fig. 3:**
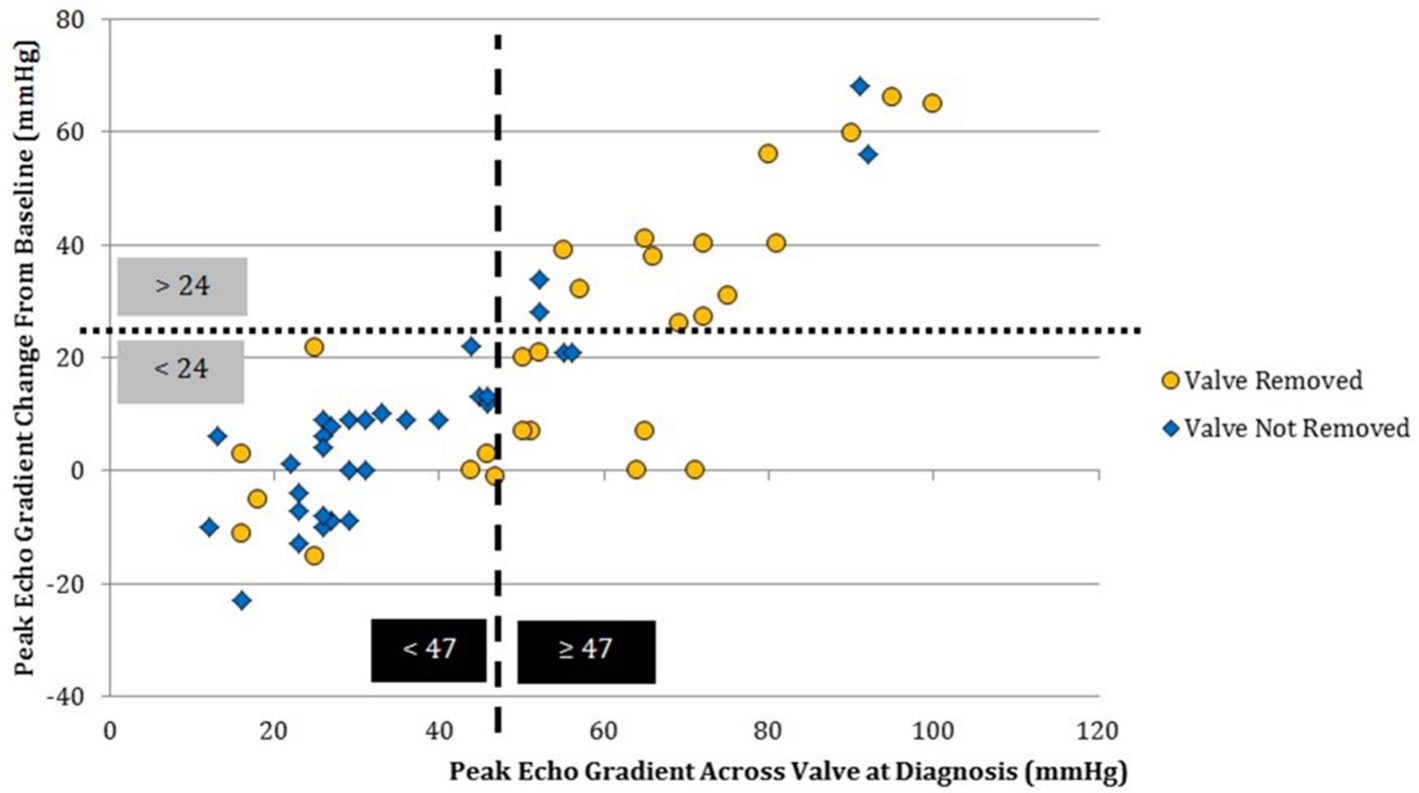
CART analysis inflection points. Blue diamonds represent patients whose *Melody* Valve was not explanted. The yellow circles represent patients whose valves were removed. The dotted lines are drawn at the inflection points identified by CART analysis (47 mmHg for the peak gradient across the RVOT at diagnosis and 24 mmHg for the change in peak gradient compared to baseline)

The rates of *Melody* TPV removal varied greatly by institution, ranging from 27% at Institution A to 64% at Institution D (Supplemental Table S1). Institutions B and C had similar rates of valve removal (40 and 44%, respectively). The most common reason for valve removal was valve stenosis, followed by presence of vegetations on echocardiography and surgeon preference (Table 4). Three patients in the explant group had pulmonary septic emboli and all three also had significant conduit stenosis. There were no cases of RVOT aneurysm formation. Two patients in the explant group had severe *Melody* regurgitation and both also had significant conduit stenosis. Patients were intubated, on inotropic support, or both prior to *Melody* TPV explantation in only 5 out of the 27 cases. The time from diagnosis of endocarditis to valve explantation ranged from 2 to 265 days (mean 50.2 days, median 24.5 days). The degree of valve stenosis (echocardiogram) was strongly associated with the decision to explant the valve. CART analysis demonstrated two important parameters associated with valve removal: a peak gradient greater than or equal to 47 mmHg by echocardiography at IE diagnosis and an increase of greater than 24 mmHg compared with the peak gradient from the most recent echocardiogram before diagnosis (Fig. 3). The odds ratio (OR) for explantation for valves with a total peak gradient ≥ 47 mmHg at diagnosis was 10.6 (*p* < 0.001). The OR for valves with a change in gradient greater than 24 mmHg at IE diagnosis compared to their baseline was 6.7 (*p* = 0.01) (Fig. 2).

**Table 4:** Reasons for *Melody* TPV removal

Reasons for valve removal	Cases, *N* (% of total)
Stenosis	16 (59%)
Stenosis alone	4 (15%)
Stenosis with vegetations	3 (11%)
Stenosis and surgeon preference	3 (11%)
Stenosis with right ventricular dysfunction	2 (7%)
Stenosis and insufficiency	1 (4%)
Stenosis with insufficiency and right ventricular dysfunction	1 (4%)
Vegetations	8 (30%)
Surgeon preference	6 (22%)
Concern for inadequate response to antimicrobials	3 (11%)
Recurrent endocarditis	1 (4%)
Total	27

Although CART analysis identified parameters strongly predictive of valve removal, there were patients with these covariates who were treated successfully with IV antimicrobials alone. Conversely, four patients without significant valve stenosis or a significant increase in gradient across the valve compared to baseline underwent surgical explantation of the *Melody* TPV. Further details regarding these outliers are included with the supplemental materials (Supplemental Information: CART Analysis Outliers).

The time to diagnosis of IE from *Melody* TPV implantation was similar among the explanted and non-explanted groups. The residual transcatheter RVOT gradient immediately after *Melody* TPV placement was higher in the explanted group, but the difference was not statistically significant. All patients received adequate antimicrobial coverage, although some patients did receive coverage that was broader than recommended by the American Heart Association (AHA) guidelines. There was no statistically significant difference between the explanted and non-explanted groups for patients who had vegetations on echocardiography, infections caused by streptococcal species, or the number of pre-stents. Of the seventeen cases caused by *Staphylococcus aureus*, ten were explanted. This difference did not meet statistical significance (*p* < 0.07). Of 24 patients with vegetations noted on echocardiography, 10 (42%) were successfully treated with IV antimicrobials only (Supplemental Figure). Four of the five IE patients with *Contegra* conduits prior to *Melody* TPV implantation underwent valve explantation.

## Discussion

In this multicenter study of 663 *Melody* TPV implantations, the overall incidence of subsequent IE was 9.9% (66 cases). Twenty-seven of those cases resulted in valve removal, while thirty-nine cases were treated with IV antimicrobials without valve removal. 44% of patients diagnosed with IE after *Melody* TPV were successfully treated with IV antimicrobials without recurrence or valve explantation. Recurrent infections were documented in 10% of patients with initial infections that were successfully treated with antimicrobials. Two patients had one additional episodes of endocarditis, both treated successfully with IV antimicrobials with no recurrence after 2 years of follow-up. These findings suggest that treatment with medical therapy alone is a reasonable approach in many cases of *Melody* TPV endocarditis. In comparison, although surgically placed valved pulmonary conduits are reported to have a lower incidence of endocarditis (2.9%) [14], recent studies show a larger proportion (58–70%) of valved conduits requiring replacement when conduit endocarditis does occur [14, 15].

Comparing patients who underwent surgical valve removal to those who did not, there was no statistically significant difference in residual RVOT gradient (immediately after *Melody* TPV implantation) nor in the number of pre-stents. Prior studies have reported that a higher residual RVOT gradient after *Melody* TPV implantation and a higher number of pre-stents are associated with a greater risk of developing endocarditis [4, 7]. Our findings suggest that those with a higher residual RVOT gradient and a higher number of pre-stents are not necessarily at higher risk of requiring valve removal should they develop endocarditis. Prior studies have also shown that infection with non-streptococcal species is more likely to result in explantation [7], but we found that there was no statistically significant difference in rates of explantation for streptococcal and non-streptococcal species. This finding may be due to a lack of power.

In our cohort, patients who underwent valve removal were more likely to have moderate or severe stenosis across the *Melody* valve at the time of diagnosis and a higher change in gradient compared to baseline echocardiography. Although not statistically significant, we found that cases of IE caused by *Staphylococcus aureus* (both MSSA and MRSA) had higher rates of explantation (58% *S. aureus* vs. 32% in non-staphylococcal). Two out of the four deaths were in cases of *Melody* IE caused by *Staphylococcus aureus*. These findings did not reach statistical significance, likely owing to the small number of cases. Nevertheless, the fact that two out of the four deaths related to *Melody* IE in our cohort were in patients with cultures positive for MSSA suggests that clinicians should have a high level of concern when endocarditis is caused by *S. aureus*. Therefore, a high-valve gradient at the time of *Melody* TPV endocarditis diagnosis and MSSA involvement might identify cases which should undergo immediate valve removal.

**Fig. 4:**
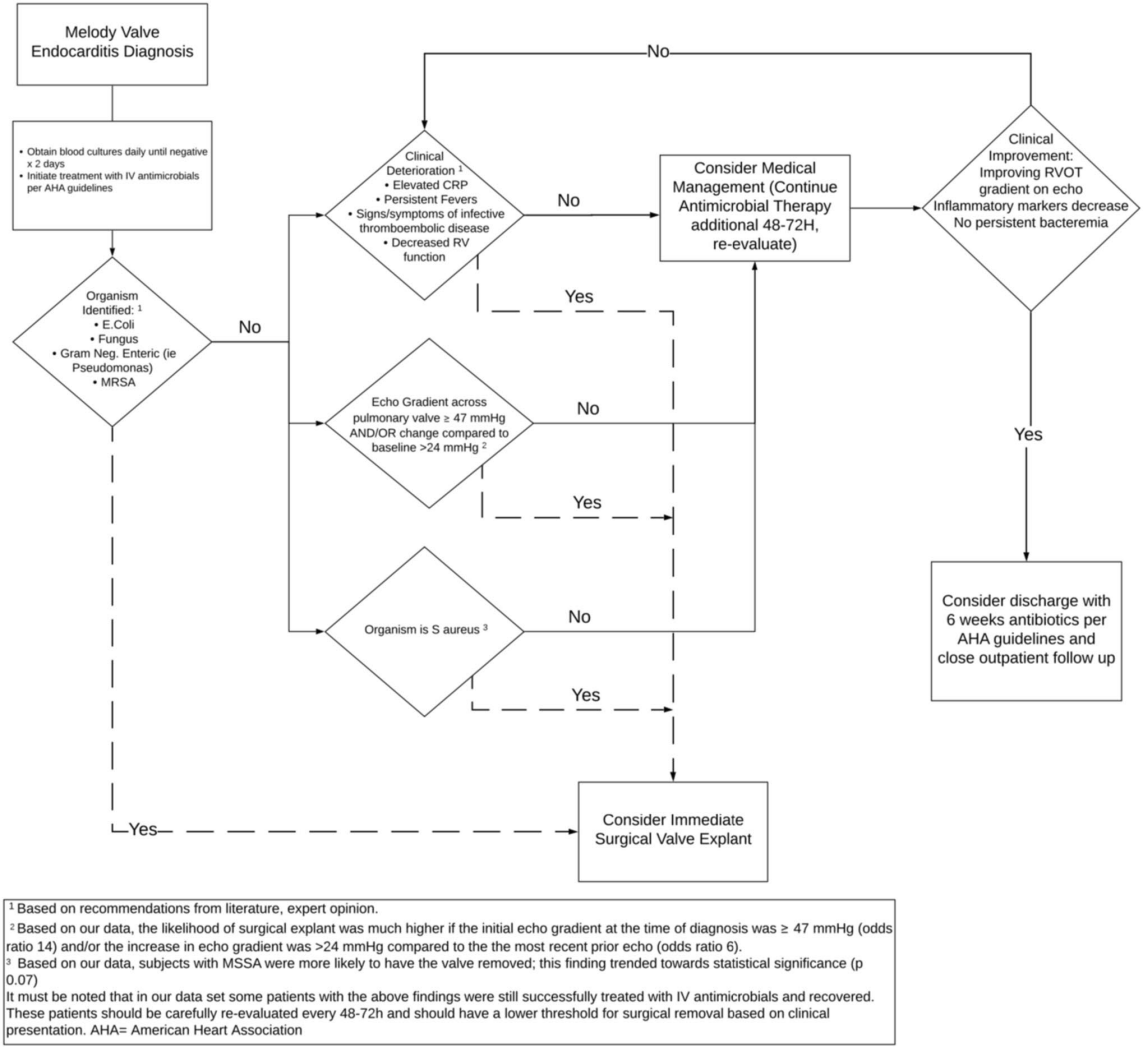
Proposed clinical framework

In this study, four cases (6%) of *Melody* TPV endocarditis resulted in mortality—one in the explant group and three in the non-explant group. Our mortality rate of 6% is similar to the mortality rate of 6.7% in patients with CHD with IE not necessarily associated with a *Melody* Valve [13]. By comparison, overall mortality from IE in adults, not associated with *Melody* Valves, has been reported to be between 18 and 23% [11, 12]. Some cases of *Melody* TPV IE result in extremis and surgery is deemed necessary for infection control. Using intubation status and the presence of inotropic support as indicators of shock and critical illness, only a minority of patients required valve explantation for these reasons (only 5/27 cases were intubated, on inotropic support, or both prior to explantation). In other cases, the decision to explant the valve may be based on institutional or surgeon preference. There was significant institutional variation in the proportion of *Melody* TPV endocarditis cases that were treated with surgical removal (ranging between 27 and 64% among the four centers). Our analysis identified factors associated with valve removal versus medical treatment for the entire cohort, regardless of institution. While acknowledging the limitations of a retrospective study and the potential for institutional bias, we have constructed a framework for evaluation and decision-making in these patients within our institutions (Fig. 4). This framework is based upon factors most strongly associated with valve removal.

A significant limitation in this retrospective study is the lack of standardized medical practice for diagnosis and medical versus surgical treatment of IE. We cannot say with certainty which cases treated with explantation could have been successfully treated with solely IV antimicrobial therapy. Conversely, perhaps some patients with endocarditis recurrence and/or valve dysfunction that were treated with IV antimicrobial therapy alone may have benefited from explantation of the *Melody* TPV. A definitive diagnosis of *Melody* TPV endocarditis using tissue culture/histology could only be made in those whose valve was explanted. A vegetation was only seen by echo in 36% of cases; however, it is often difficult to visualize vegetations by echocardiography so there may have been patients who had vegetations that were not visualized. As this is a retrospective study, we acknowledge that the diagnosis of endocarditis was not standardized and was at the discretion of clinicians at each site. This adds an inherent subjectivity to the determination of IE. Additionally, there currently is no evidence-based standard treatment protocol for *Melody* TPV endocarditis.

We attempted to identify trends based on which patients underwent surgical removal and those that did not. Since the initial decision for surgery versus medical treatment was not standardized, it is unclear to what extent our findings simply represent institutional variations in practice. Additionally, the relatively few cases included in this study prohibited adequate power to determine whether the lack of statistical significance was due to a type 2 error alone.

## Conclusion

Not all patients who develop IE of the *Melody* valve require explantation. In our multicenter experience, 44% of patients with *Melody* valve endocarditis were successfully medically treated without valve explantation or recurrence. A peak gradient ≥ 47 mmHg by echocardiography at time of endocarditis diagnosis and an increase in peak gradient across the valve of > 24 mmHg on echocardiography at time of endocarditis diagnosis were associated with valve removal. Cases of IE caused by *Staphylococcus aureus* more often resulted in surgical removal and mortality. Our findings in this large multicenter study of 663 *Melody* TPV procedures has resulted in an internal institutional framework for clinical decision-making regarding valve removal versus medical treatment. Further studies are needed to better characterize clinical and echocardiographic metrics predictive of poorer clinical outcomes and to better clarify indications for urgent explantation versus a trial of medical management alone. Based on our retrospective data, our proposed framework should be studied prospectively in a controlled trial.

## Supplementary Information

Below is the link to the electronic supplementary material.

Supplementary file1 (DOCX 331 kb)
