# Supplementary material for: Selective Valve Removal for Melody Valve Endocarditis: Practice Variations in a Multicenter Experience
Source: Pediatr Cardiol. 2021 Dec 11;43(4):894–902. doi: 10.1007/s00246-021-02801-z (PMC9005409; doi:10.1007/s00246-021-02801-z)
Supplement: Supplementary file 1 — Supplementary file1 (DOCX 331 kb) [file 246_2021_2801_MOESM1_ESM.docx]

#### Supporting Material

#### Supplemental Figure


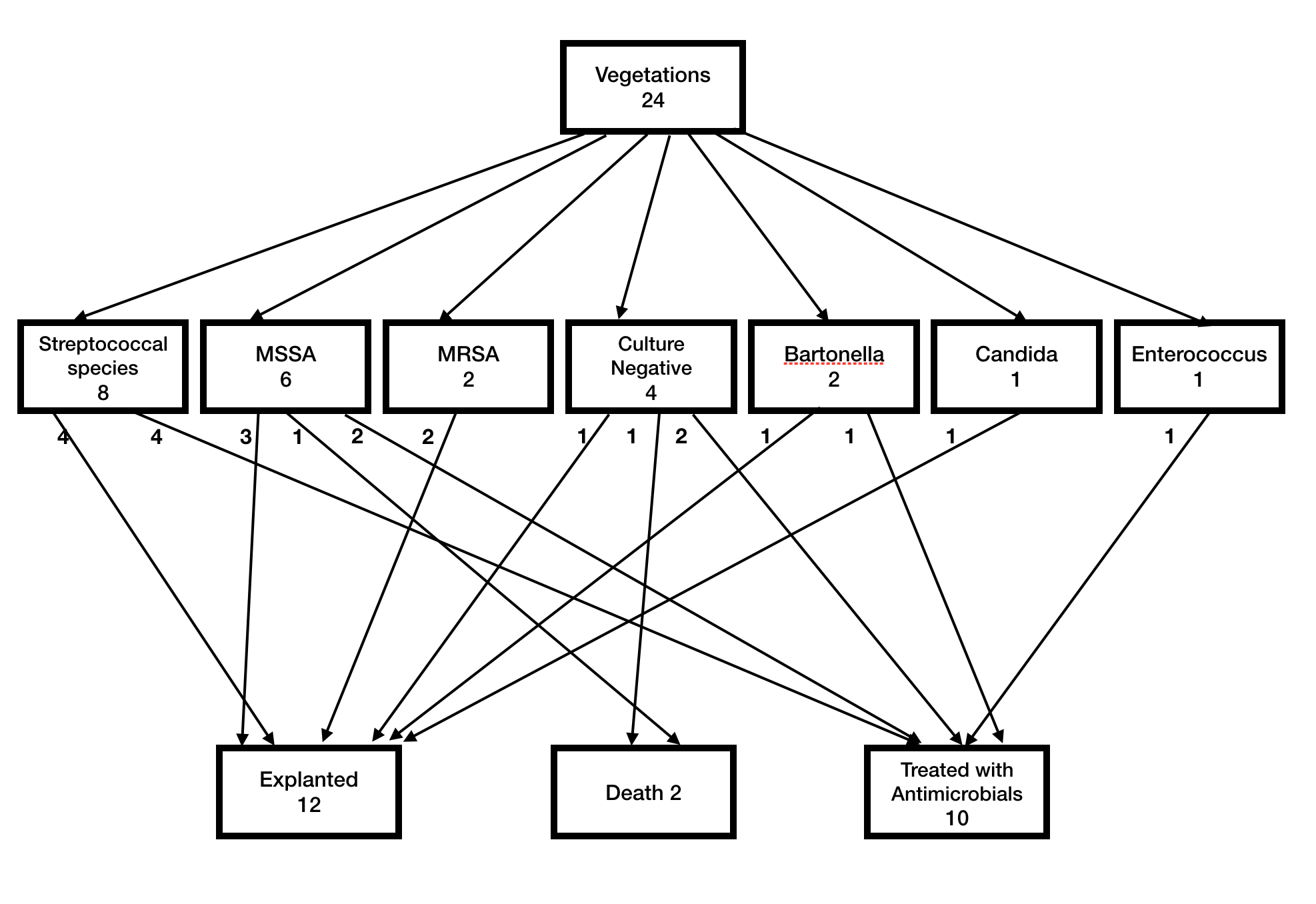


Supplemental figure 1. Outcomes in patients with vegetations identified on echocardiography. There were a total of 23 patients that were noted to have vegetations on echocardiography. The patients with vegetations had infective endocarditis caused by streptococcal species (8), MSSA (6), MRSA (2), Bartonella (2), candida (1), enterococcus (1) and culture negative (4). The outcomes (explantation, death, and successful treatment with IV antimicrobials) are detailed for each of the patients in each organism category.

#### Supporting Table 1. Rates of Melody explantation by institution

| **Institution** | **Proportion Explanted (%)** |
| --- | --- |
| **A** | 4/15 (27%) |
| **B** | 10/28 (36%) |
| **C** | 4/9 (44%) |
| **D** | 9/14 (64%) |

#### Supporting Information: Infective Endocarditis Recurrences

The first patient of those with one recurrent IE episode was initially treated medically for viridans group streptococcus endocarditis with full recovery. One year later, this patient presented with fever, chills and malaise. Blood cultures were negative but he had new valvar regurgitation and was diagnosed with acute infective endocarditis. He later developed worsening stenosis of the valve (peak gradient of 52 mmHg) and the valve was removed due to stenosis and insufficiency. The second patient was initially treated medically for *Haemophilus parainfluenzae* endocarditis. Five years later, this patient had *Cardiobacterium hominis* endocarditis requiring valve explantation and died during the same hospitalization. The third patient, initially treated for culture negative endocarditis with full recovery, presented 1.2 years later with group A Streptococcus bacteremia. This patient was successfully treated with IV antimicrobials with no further recurrence or significant valve dysfunction (follow up > 2 years from second IE episode). The fourth patient was treated for *Enterococcus faecalis* endocarditis with IV antimicrobials with full recovery. Two years later, he presented with *Streptococcus gallolyticus* endocarditis and was again treated with IV antimicrobials without further recurrence (follow up >4 years from second IE episode).

The first patient of those with two recurrent IE episodes had three episodes all caused by streptococcal species and treated with IV antimicrobials. The first episode was caused by nutritionally variant streptococci (*abiotrophia/granulicatella*). A second episode 2.2 years later was caused by *Streptococcus mutans.* The third episode, which occurred 3.2 years after the second episode, was caused by a viridans streptococcus (not further speciated). The *Melody* TPV remains in place (follow up duration 2.7 months from the third episode of IE). The second patient had removal of the *Melody* TPV after endocarditis with methicillin sensitive *Staphylococcus aureus* (MSSA) and placement of a homograft conduit. The patient had a second *Melody* TPV implanted within the homograft conduit 3.2 years later. Two months after implantation of this second *Melody* TPV, the patient developed MSSA endocarditis which was treated with IV antimicrobials. Three years later, the patient had a third episode of MSSA endocarditis, and the *Melody* TPV was explanted.

#### Supporting Information: CART Analysis Outliers

Though CART analysis identified parameters strongly predictive of valve removal, there were patients with these covariates who were treated successfully with IV antimicrobials alone. Four patients treated successfully with IV antimicrobials had both a gradient >47 mmHg at diagnosis and a change from baseline >24 mmHg. Three of the four had no further episodes of endocarditis or valve dysfunction. Streptococcal species were identified in two of these cases, and the other was culture negative. The fourth patient was initially treated for streptococcal endocarditis with full recovery. One year later this patient had culture negative endocarditis and valve explantation. Two patients with a gradient >47 mmHg (but change in baseline <23 mmHg) whose causative organisms were MSSA and *Enterococcus faecalis* were treated successfully with IV antimicrobials.

Conversely, four patients without significant valve stenosis or a significant increase in gradient across the valve compared to baseline underwent surgical explantation of the *Melody* TPV. Three of the four patients were from Institution D (which had the highest rate of explantation). One patient with culture negative IE had a peak gradient of 18 mmHg across the valve which was a decrease from their baseline of 23 mmHg. This was their second episode of endocarditis. The second patient had culture negative IE, a peak gradient of 16 mmHg across the valve, and an increase of only 3 mmHg compared to baseline. This patient had persistent vegetations and the valve was explanted. The third patient also had a peak gradient of 16 mmHg across the valve, improved from a gradient of 27 mmHg baseline. This patient had viridans group streptococcus IE and the reason for valve removal was cited as “surgeon preference”. The fourth patient had a peak gradient of 25 mmHg across the *Melody* TPV*,* which was a decrease from baseline (40 mmHg), but this was the third episode of *Melody* TPV *IE* and second *Melody* TPV.
